# Correction: Embryonic and postnatal neurogenesis produce functionally distinct subclasses of dopaminergic neuron

**DOI:** 10.7554/eLife.99599

**Published:** 2024-05-10

**Authors:** Elisa Galliano, Eleonora Franzoni, Marine Breton, Annisa N Chand, Darren J Byrne, Venkatesh N Murthy, Matthew S Grubb

**Keywords:** Mouse

Galliano E, Franzoni E, Breton M, Chand AN, Byrne DJ, Murthy VN, Grubb MS. 2018. Embryonic and postnatal neurogenesis produce functionally distinct subclasses of dopaminergic neuron. *eLife* **7**:e32373. doi: 10.7554/eLife.32373.

Published 20 April 2018

Upon recent checks of our electrophysiology solutions, we discovered that the composition of artificial cerebrospinal fluid (ACSF) described in this article contained two inaccuracies. Our ACSF contained 2.5 mM KCl, not the 5 mM originally reported in the article, and 15 mM d-glucose rather than the originally reported 20 mM. We sincerely apologise for this oversight and have now made the necessary corrections to the ‘Acute slice electrophysiology’ part of the ‘Materials and methods’ section of the manuscript. These minor corrections apply to all slice electrophysiology experiments carried out in the paper, and are equal across both cell subtypes studied. They therefore have no bearing on any of the results reported or conclusions drawn in the original version. All authors have expressed their unanimous agreement with the correction text.

Corrected text:

Horizontal slices (300 µm thick) of the olfactory bulb were cut using a vibratome (VT1000S, Leica) and maintained in ACSF containing (in mM): 124 NaCl, 2.5 KCl, 1.25 Na_2_HPO_4_, 2 MgSO_4_, 2 CaCl_2_, 26 NaHCO_3_ and 15 d-Glucose, bubbled with 95 % O_2_ and 5 % CO_2_ for >1 hr before experiments began.

Original text:

Horizontal slices (300 µm thick) of the olfactory bulb were cut using a vibratome (VT1000S, Leica) and maintained in ACSF containing (in mM): 124 NaCl, 5 KCl, 1.25 Na_2_HPO_4_, 2 MgSO_4_, 2 CaCl_2_, 26 NaHCO_3_ and 20 d-Glucose, bubbled with 95% O_2_ and 5% CO_2_ for >1 hr before experiments began.

The article has been corrected accordingly.

